# Inequality and inequity in eye health

**Published:** 2016

**Authors:** Johannes Trimmel

**Affiliations:** Director: Policy and Advocacy, International Agency for the Prevention of Blindness (IAPB), Vienna, Austria. **jtrimmel@iapb.org**

**Figure:**
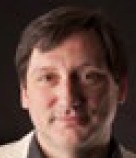
Johannes Trimmel

According to the 2010 Global Burden of Disease (GBD) study, the global prevalence of blindness (age-standardised) has declined from 0.60% in 1990 to 0.47% in 2010.^1^ This seems to indicate that an increasing number of people have access to good eye health services.

However, this improvement is not equally distributed within and across nations. The GBD study also showed that 60% of blindness worldwide is among women, underlining that gender equity in eye health has not yet been achieved.

There are several other studies which show how inequitable access to eye health services is worldwide. A recent assessment of avoidable blindness and visual impairment in seven Latin American countries concluded that the prevalence of blindness and moderate visual impairment was concentrated among the most socially disadvantaged, and that cataract surgical coverage and cataract surgery optimal outcome were concentrated among the wealthiest.^2^ The same study showed that unoperated cataract remained the most common cause of blindness in Argentina, despite the high national cataract surgical rate (CSR) of 5,935 cataract operations per million population per year. A 2010 study in Gujarat, India concluded that, despite an even higher reported CSR of 10,000, cataract remained the predominant cause of blindness and visual impairment and blindness remained a significant problem among the elderly.^3^ A systematic review of barriers to cataract surgery in Africa^4^ (which involved reviewing 86 articles, including 12 RAAB, 10 quantitative and 5 qualitative studies) showed variability in the study outcomes. In the RAAB studies, barriers related to awareness and access were more commonly reported. Other studies reported cost as the most common barrier. Some qualitative studies tended to report community and family dynamics as barriers to cataract surgery. Overall, the systematic review found that the CSR was lower in females in 88.2% of the studies. These major barriers point to underlying factors of unequal access: illiteracy and low educational levels, poverty and economic hardship, no physical access (distance), and the socio-cultural situation.

**Figure:**
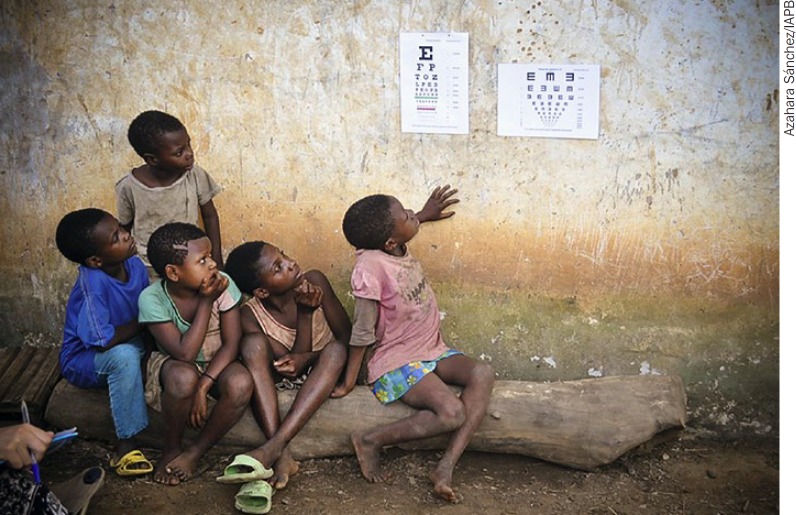
People living in rural areas or in poverty are often unable to access eye care, even when it is available free of charge. It is important to bring eye care closer to these communities, for example by offering visual acuity screening in the community. CAMEROON

**INEQUALITY AND INEQUITY: WHAT ARE WE TALKING ABOUT?**

**Figure:**
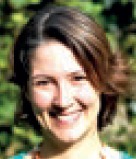
Elmien Wolvaardt Ellison

Editor: *Community Eye Health Journal*, International Centre for Eye Health, London, UK.

According to the World Health Organization, inequalities in health can exist for various reasons, some of which are biological (e.g. a higher incidence of cataract in people over 60 years of age). If these inequalities are **avoidable**, however – e.g. if services were made more affordable – then they are better described as **inequities**, a word which captures the unfairness of the situation.

Equal provision of eye health does not create equity: it is important to ensure that eye care provision is proportional to need (see Figure 1 on page 3). For example, women are often much more likely than men to have age-related cataract. An equal number of operations for women and men would therefore be **inequitable**, as women's needs are greater.

While, increasingly, data on eye health provision are collected separately by gender, age and economic situation (known as ‘disaggregation of data’), there is less information available for ethnic minorities, migrants and people with disabilities. In the United Kingdom, people from black and minority ethnic communities are at greater risk of some of the leading causes of sight loss, and adults with learning disabilities are 10 times more likely to be blind or partially sighted than the general population.^5^

The World Report on Disability^6^, jointly produced by the World Health Organization (WHO) and the World Bank, states that the affordability of health services and transportation are two main barriers for people with disabilities to access health services. In low-income countries, 36% of non-disabled females and 40% non-disabled males could **not** afford the visit to the health service provider, compared to 61% (female) and 59% (male) of disabled people. A recent study from Sightsavers on data disaggregation by disability in India and Tanzania^7^ showed that, despite the eye health programmes being open to all, the level of access of people with disabilities varied greatly.

As these examples show, there are many dimensions to inequity. Inequity can be understood as a reflection of multidimensional poverty which, besides income poverty, includes poor health, low levels of education, lack of water and sanitation, an unhealthy or unsafe residential environment, insecurity and violence, social exclusion, lack of participation, disempowerment, a lack of self-esteem, and more.

The multidimensional understanding of poverty is reflected in the Sustainable Development Goals (SDGs, see page 4). Adopted by the UN General Assembly in September 2015, they comprehensively address the economic, social and environmental dimension of sustainable development. There is a strong focus on tackling the systemic issue of inequity and a promise to ‘leave no one behind’. The World Health Organization action plan called Universal Eye Health: A Global Action Plan 2014–2019^8^ has established universal access and equity, human rights, and empowerment of people with visual impairment as core principles.

## What can we do?

To tackle inequities in eye health, a number of measures can be taken. First of all, as eye care providers we should commit to providing eye health services of the same quality for everybody, irrespective of age, gender, wealth, ethnicity, place of residence, education or disability status. Just as important, as individuals we should treat everyone equally on a personal level: everybody turning up at an eye health clinic or hospital should enjoy the same level of interest, respect and support. As authors we recommend awareness training of staff members and setting quality standards that are monitored regularly.

**Figure 1.**
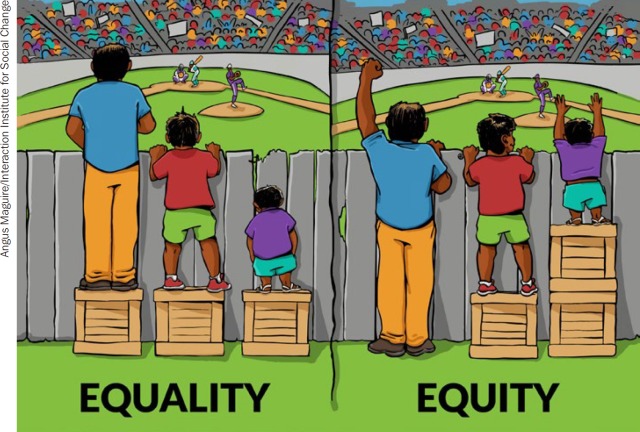
Providing equal eye care services is not enough – equity is only achieved when the eye care services meet the needs of different groups of patients

Eye care units can be made more accessible for people with disabilities as outlined in the CBM Guide ‘Inclusion made easy in eye health programmes’.^9^

Eye care providers are also employers and can support inclusion and diversity by recruiting a wide spectrum of staff members: those with or without disabilities, from both genders (and transgender), all sexual orientations, and all population groups. This will not only support communication with patients, but also help to increase understanding and awareness among staff members.

## A change in perspective

However, offering equal eye health services to everyone will not by itself lead to equity in eye health services. Equal services will only be effective at reducing inequity if every person in the community has the same starting point (Figure 1). Evidence and life experience show that this is not the case, and that an equality of service provision alone is insufficient to promote fairness and justice.

What is needed to address inequity in eye health effectively is a change of perspective. Rather than putting the eye care service unit at the centre of planning and action, it is necessary to look at eye health programmes from the point of view of the person needing eye health services and what they need to enjoy a full and healthy life. The best eye clinic will not deliver what people need when there are barriers that prevent them from arriving there!

Engagement at the community level is very much needed. Many of the barriers identified – ignorance, lack of awareness, cultural traditions, to name a few- need to be effectively addressed at family and community level. As these barriers are not specific to eye health, there needs to be either partnerships with other community-based services (primary health care and community development in general) or a policy framework which addresses these issues effectively. This is particularly relevant for eye care providers who implement community outreach activities.

In any community initiative, participation and empowerment are key. By specifically addressing people, families and groups who are socially excluded in local communities we can tackle inequity and help to change the behaviour of mainstream society.

## A helpful policy framework

The Sustainable Development Goal on health calls for efforts to ensure healthy lives and wellbeing at all ages. To achieve this, a number of political decisions need to be taken and policy choices made. Besides lack of knowledge and awareness, cost is the most prominent factor leading to inequity in eye health.

Universal health coverage and social insurance (or cost coverage) schemes are currently being put in place in many countries to help cover the cost of health care. These must be designed to actively and effectively include people from disadvantaged and poor populations. Simply reviewing whether or not these groups are equally included is not enough: there is a high likelihood that poorer people (understood in terms of multidimensional poverty) do not seek the services they need due to ignorance, fear, lack of means for transport, and other reasons. Positive and pro-active (affirmative) action is required to ensure that upcoming cost-coverage schemes are not only effective for the educated and socially included, but also reach out to the poorest. Affirmative action is not discriminatory, as the UN Convention on the Rights of Persons with Disabilities states in Article 5 (4): ‘Specific measures which are necessary to accelerate or achieve de facto equality of persons with disabilities shall not be considered discrimination under the terms of the present convention.’

A popular quote these days is: ‘If you can't measure it, you can't manage it’. While this rightly can be questioned – as there are many qualities in life not easily measurable – evidence also shows that it is very hard to achieve political support for addressing inequities in eye health unless there is reliable data. Accordingly, the SDGs requires that high-quality, timely and reliable data – disaggregated by income, gender, age, race, ethnicity, migratory status, disability, geographic location (and other characteristics relevant in national contexts) – is made available. This should be standard for the eye health sector as well.

## Conclusion

Tackling unequal access to eye health services and inequity in eye health requires a people-based view and an approach that reaches far beyond service provision. Moving outside the eye health sector (or silo) is essential in order to reduce inequity in eye health. The current international development frameworks (see article on page 4), which also put a strong emphasis on domestic resource mobilisation, provide an excellent framework which needs to be used.
